# The First Report of a Virulent Newcastle Disease Virus of Genotype VII.2 Causing Outbreaks in Chickens in Bangladesh

**DOI:** 10.3390/v14122627

**Published:** 2022-11-25

**Authors:** Mohammed Nooruzzaman, Ismail Hossain, Jahan Ara Begum, Moktader Moula, Shamsul Arefin Khaled, Rokshana Parvin, Emdadul Haque Chowdhury, Mohammad Rafiqul Islam, Diego G. Diel, Kiril M. Dimitrov

**Affiliations:** 1 Department of Pathology, Faculty of Veterinary Science, Bangladesh Agricultural University, Mymensingh 2202, Bangladesh; 2 Department of Population Medicine and Diagnostic Sciences, College of Veterinary Medicine, Cornell University, Ithaca, NY 14853, USA; 3 Nourish Poultry and Hatchery Limited, 39 Sonargaon Janapath, Dhaka 1230, Bangladesh; 4 Texas A&M Veterinary Medical Diagnostic Laboratory, 483 Agronomy Rd, College Station, TX 77843, USA

**Keywords:** Newcastle disease virus, genotype VII.2, chickens, pathogenicity, Bangladesh

## Abstract

Newcastle disease (ND) is endemic in poultry in Bangladesh. We performed genotypic and pathotypic characterization of four ND virus (NDV) isolates from recent outbreaks in broiler chickens in Bangladesh during the period of 2020–2021. Phylogenetic analysis based on the complete fusion protein gene coding sequences classified the viruses into NDV class II genotype VII.2 together with viruses from Indonesia isolated between 2014 and 2021 and a single 2020 Indian isolate. Pathogenicity testing using the intracerebral pathogenicity index in day-old chickens and mean embryo death time in embryonating chicken eggs revealed that the Bangladeshi isolates are velogenic. Inoculation of 35-day-old chickens with two NDV isolates (LT67 and N5) resulted in 100% morbidity by 3 days post inoculation (DPI), and all birds succumbed to infection by 7 DPI. Massive hemorrhages, congestion and necrotic lesions were observed in different visceral organs, which were typical for infection with a velogenic viscerotropic pathotype of NDV. At microscopic examination, tracheitis, severe pneumonia, focal proventriculitis, transmural enteritis, focal myocarditis, severe congestion and necrosis in kidneys, and lymphoid depletion in lymphoid tissues were found. Our study reports the first outbreak of the panzootic genotype VII.2 NDV in poultry in Bangladesh and documents a possible recent re-introduction of this NDV genotype from Southeast or East Asia. This study further provides viral distribution and epidemiological data that can facilitate the effective control of NDV.

## 1. Introduction

Newcastle disease (ND) is a highly contagious and fatal viral disease of poultry causing high economic burden worldwide. The disease is caused by Newcastle disease virus (NDV, or avian paramyxovirus type 1), a species within the genus *Avian orthoavulavirus 1* of the family *Paramyxoviridae* [1]. The virus is enveloped and has a negative-sense, single-stranded RNA genome of approximately 15.2 kb coding for six structural proteins: nucleocapsid protein (NP), phosphoprotein (P), matrix protein (M), fusion protein (F), haemagglutinin-neuraminidase (HN), and large RNA-dependent polymerase (L). The HN protein recognizes and attaches to sialic acid receptors on the surface of permissive cells and mediates the fusion activity of the F protein at the cell membrane for release of the nucleocapsid complex into the cytoplasm [2].

Strains of NDV vary in virulence and can be divided into pathotypes based on their pathogenicity in chickens, listed in increasing order of virulence: lentogenic (avirulent or of low virulence), mesogenic (of moderate virulence), and velogenic (highly virulent) [3]. The velogenic strains can be further divided into viscerotropic and neurotropic depending on the predominant clinical signs and lesions they cause in the gastrointestinal tract or central nervous system of infected chickens, respectively [4]. The molecular basis of pathogenicity of NDV strains is the amino acid residues motif at the fusion protein cleavage site and the ability of specific cellular proteases to cleave this protein. Virulent NDV strains possess multiple basic amino acid residues (arginine or lysine) at the fusion protein cleavage site which can be cleaved by ubiquitous host proteases leading to fatal systemic infection, while lentogenic strains possess monobasic cleavage site, that can only be recognized by the trypsin-like proteases present in the respiratory and gastrointestinal tracts [5,6].

NDV has wide genetic diversity and is phylogenetically divided into two classes, class I and class II [7]. Class I viruses belong to a single genotype, are avirulent, and mostly detected in aquatic wild birds and live bird market samples [8,9]. Viruses from class II are classified into at least 21 genotypes (I-XXI) and many sub-genotypes [10], are mostly virulent, and are responsible for outbreaks in both domestic poultry and wild birds worldwide [11,12].

Among the different genotypes of class II NDV, genotype VII has a wide geographic distribution and is one of the current dominant genotypes worldwide. Genotype VII viruses are considered to have emerged in South-East Asia during the 1980s and spread across all continents except North America and Australia. Viruses of genotype VII are divided into three sub-genotypes, VII.1.1, VII.1.2 and VII.2. Genotype VII.1.1 encompasses viruses that emerged around 1985 in the Far East and rapidly spread to Asia, the Middle East, Europe, and Africa, causing the fourth ND panzootic. Genotype VII.2 viruses emerged in Indonesia and Malaysia between 2005 and 2010, further spreading to Central and East Asia, the Middle East, Europe, and Africa, and are responsible for the ongoing fifth ND panzootic [13,14,15,16,17,18,19,20,21].

The first report of an ND outbreak in Bangladesh was published in 1981 [22]. Currently, ND is endemic in the country with reports of regular field outbreaks [23,24,25,26]. Genotypic and pathotypic studies reported circulation of velogenic NDV belonging to at least two genotypes, genotype XIII.2 in chickens [27,28,29] and genotype XXI.1.2 in pigeons [30]. As ND outbreaks are ongoing, the aim of this study was to investigate viruses causing recent outbreaks in broiler chickens in Bangladesh and characterize them molecularly, phylogenetically, and pathotypically. Here, we provide the first report of the circulation of genotype VII.2 velogenic NDV in Bangladesh.

## 2. Materials and Methods

### 2.1. Outbreaks

A total of four Newcastle disease suspected outbreaks that occurred in 2020 and 2021 in meat type chickens were investigated at the Department of Pathology of the Bangladesh Agricultural University. The affected farms were two broiler breeder and two commercial broiler farms from three different districts of Bangladesh: Mymensingh, Panchagarh, and Gazipur (map with locations provided in Supplementary Figure S1). All flocks had previously received vaccination against NDV. In all flocks, there was sudden onset of high morbidity with lethargy and respiratory distress. While the mortality in the breeder operations was low, the observed mortality rate in the broiler farms was moderate (Table 1). One of the breeder flocks (N4) was in the laying stage and birds showed a sudden and significant drop (50%) in egg production, which never reached to pre-outbreak levels after recovery. There was no available information of whether other farms in the district were experiencing similar signs. Sick or dead birds were submitted to the Department of Pathology for investigation. At necropsy, hemorrhages in the trachea, proventriculus, intestine, and cecal tonsils were found consistently in all birds. The breeder flock at laying stage showed hemorrhages in egg follicles and egg peritonitis. Detailed information on the farms is presented in Table 1.

### 2.2. Sample Collection and Virus Isolation

At necropsy, pooled respiratory tissues (lungs and trachea) were collected in sterile tubes for virus isolation. A 20% (*w*/*v*) tissue homogenate was prepared using phosphate buffered saline supplemented with gentamicin (10 mg/mL). The homogenate was clarified by centrifugation at 3000 rpm for 10 min. Supernatant (200 μL) from each tissue homogenate was inoculated into 10-day-old embryonating chicken eggs (ECEs) via the allantoic cavity route. Eggs were incubated at 37 °C and candled for vitality daily. Eggs with dead embryo and all eggs at the end of the 7-day incubation period were chilled at 4 °C. The allantoic fluids from inoculated ECE were harvested and subjected to the hemagglutination (HA) assay using standard procedure [31]. The HA positive allantoic fluids were confirmed as NDV by RT-PCR as previously described [29]. In addition, all allantoic fluids were tested for influenza A virus, infectious bronchitis virus, and infectious laryngotracheitis virus using a real-time PCR panel assay [25]. The collected allantoic fluids were titrated in embryonating chicken eggs to estimate their embryo infectious dose 50 (EID_50_) [32].

### 2.3. Full Length Fusion Gene Amplification and Sequencing

Total viral RNA from the infective allantoic fluid was extracted using PureLink RNA Mini Kit (ThermoFisher Scientific, Waltham, MA, USA) following the manufacturer’s instructions. SuperScript III One-Step RT-PCR System with Platinum *Taq* DNA Polymerase was used to amplify the full-length fusion protein gene sequences using overlapping RT-PCR. The following primer pairs were used: NDVF13-F1 5′-GAC GCA ACA TGG GCT CCA RAY CTT-3′ and NDVF13-R1 5′-GGC AAA CCC TCT GGT CGT GCT YAC-3′ [27], and the newly designed primers NDVF7F2: 5′-TCA GTC GGG AGC CTW AAT AAT AT-3′ and NDVF7R2: 5′-TCA TGC TCT TGT GGT GGC T-3′. A 50 µL reaction mix was prepared containing 25 µL 2X Reaction Mix, 10 µM of each primer, 1 µL SuperScript™ III RT/Platinum^®^ *Taq* Mix and 5 µL template RNA. The following thermal profile was followed: reverse transcription at 55 °C for 30 min followed by pre-denaturation at 95 °C for 2 min and then 35 cycles of denaturation at 94 °C for 15 s, annealing at 55 °C for 30 s and extension at 68 °C for 2 min, and final elongation at 68 °C for 5 min. The RT-PCR product was subjected to electrophoresis in 1% agarose gel. The DNA bands were excised, purified, and sequenced by a commercial laboratory (Macrogen, Seoul, Republic of Korea). The raw sequence data were assembled and edited using BioEdit software (www.mbio.ncsu.edu/BioEdit/bioedit.html, accessed on 9 May 2022).

### 2.4. Evolutionary and Phylogenetic Analysis

For comprehensive phylogenetic analysis, the dataset of complete F-gene sequences (2157 sequences as of 9 May 2022) provided by the international consortium that published the current NDV classification system [10] was used (dataset deposited in GitHub at https://github.com/NDVconsortium/NDV_Sequence_Datasets, accessed on 9 May 2022). The sequences of the four studied isolates and additional sequences, submitted to GenBank after the dataset was created, were downloaded, and added to the alignment (as of 30 August 2022). All collected sequences (*n* = 2257) were aligned using Multiple Alignment with Fast Fourier Transformation (MAFFT v7.4.50) [33].

Next, a maximum-likelihood tree with 1000 bootstrap replicates was built using RaxML version 8.2.12 [34] based on the general time-reversible (GTR) model [35] (goodness of fit measured by the corrected Akaike information criterion in MEGA 7). Evolutionary rate differences among sites were modelled with discrete Gamma distribution (Γ), and the rate variation model allowed for some sites to be evolutionarily invariable (I). The RaxML tree was constructed through the CIPRES Science Gateway [36] as described previously [10]. The tree was visualized using FigTree v1.4.2 (http://tree.bio.ed.ac.uk/software/figtree, accessed on 9 May 2022). The taxa names include a Roman–Arabic numeral representing the respective genotype for each isolate, the GenBank accession number, host name (if available), country of isolation, isolate designation, and year of isolation. The criteria put forth by the NDV consortium [10] based on the phylogenetic topology and evolutionary distances between different taxonomic groups were used for sub/genotype identification.

Furthermore, we obtained all fusion gene sequences (partial or complete) of NDV from Bangladesh submitted to GenBank. A total of 45 fusion gene sequences from chickens, pigeons, and migratory birds from Bangladesh were selected for the analysis based on their common sequence region (as of 30 August 2022). The maximum-likelihood tree based on the GTR model was built using RaxML version 8.2.12 with 1000 bootstrap replicates. 

The estimates of average evolutionary distances between different genotypes of NDV and the isolates of the present study were calculated with MEGA7 [37] using the Maximum Composite Likelihood model [38]. The rate variation among sites was modelled with a gamma distribution (shape parameter = 1).

Bayesian time scaled analysis was conducted by the Bayesian Markov Chain Monte Carlo (BMCMC) method implemented in BEAST v1.10.4 [39] program utilizing a subset of all full-fusion gene sequences (*n* = 1013) of genotypes VII (sub-genotypes VII.1.1, VII.1.2 and VII.2). General time-reversible model with gamma distribution nucleotide substitution were applied (GTR + G4) [35,40]. Relaxed clock model (uncorrelated lognormal distribution) [41] with exponential growth demographic model was utilized. An input file for BEAST analysis was prepared using the Bayesian evolutionary analysis utility (BEAUTI) tool v.1.10.4 included in the BEAST package, and the sequences were annotated with year of collection. Three independent chains were run through the CIPRES Science Gateway using BEAGLE library [42] to get output of 100,000 trees from each run. Convergence was assessed in Tracer v1.7.1 program [43]. The trees from each run were combined using LogCombiner v.1.10.4 (burn in 30%), and Maximum Clade Credibility tree was generated using the Tree Annotator program v.1.10.4 from the BEAST package. The FigTree v1.4.2 tool was used for the visualization of the annotated tree.

### 2.5. Cleavage Site Analysis and Pathogenicity Testing

Analysis of deduced amino acid sequences of fusion protein cleavage site of the NDV isolates was performed using MEGA 7 software. The pathogenicity of three of the studied isolates from Bangladesh (N1, LT67, and N5, could not achieve a sufficient titer for N4) was assessed by estimating the mean death time (MDT) of embryonating chicken eggs and the intracerebral pathogenicity index (ICPI) in day-old chicks following standard procedures [31].

### 2.6. Experimental Infection in Chickens

A total of 30 layer chickens of the ISA Brown breed were raised from day-old in relative isolation under strict biosecurity measures. Feed and water were provided with *ad libitum* access. At 33 days of age, 10 serum samples were collected randomly from the birds and tested for the presence of NDV specific maternal antibodies using a commercial ELISA kit (ID Screen^®^ Newcastle Disease Indirect, IDVet, Grabels, France) as well as by hemagglutination inhibition (HI) test.

At 35 days of age, chickens were divided into three groups (group A–C) and housed separately. Chickens from group A (control, *n* = 10) received sterile PBS (0.2 mL/bird) through the intranasal and intraocular routes. Chickens from the challenge groups (B and C, *n* = 10 each) were inoculated through intranasal and intraocular routes with 10^5^ EID_50_ (0.2 mL/bird) of the infected allantoic fluids of LT67 and N5 NDV isolates. These two isolates were selected as they were obtained from two distant geographical locations. All birds were observed daily for clinical signs and mortality.

### 2.7. Necropsy and Pathological Study

Chickens that died during the observation period were subjected to necropsy immediately. In addition, all surviving birds (from control uninoculated group A) at 7 days post inoculation (DPI) were euthanized and necropsied. At necropsy, the gross pathological changes in different organs were recorded. A part of the lungs and trachea was collected aseptically in a sterile tube for virus detection by RT-PCR as described earlier [29]. In addition, tissues from the trachea, lungs, proventriculus, intestine, caecal tonsils, liver, kidney, heart, thymus, spleen, and bursa of Fabricius were collected in 10% neutral buffered formalin for microscopic examination. Fixed tissue samples were processed, sectioned, and stained with routine hematoxylin and eosin staining method [44]. The slides were examined under a photomicroscope (CX43 Olympus, Tokyo, Japan).

### 2.8. Accession Numbers

The nucleotide sequences of the four studied isolates generated in this study were deposited in GenBank (Accession no. OP378144-OP378147).

## 3. Results

### 3.1. Evolutionary and Phylogenetic Analysis

**Figure 1:**
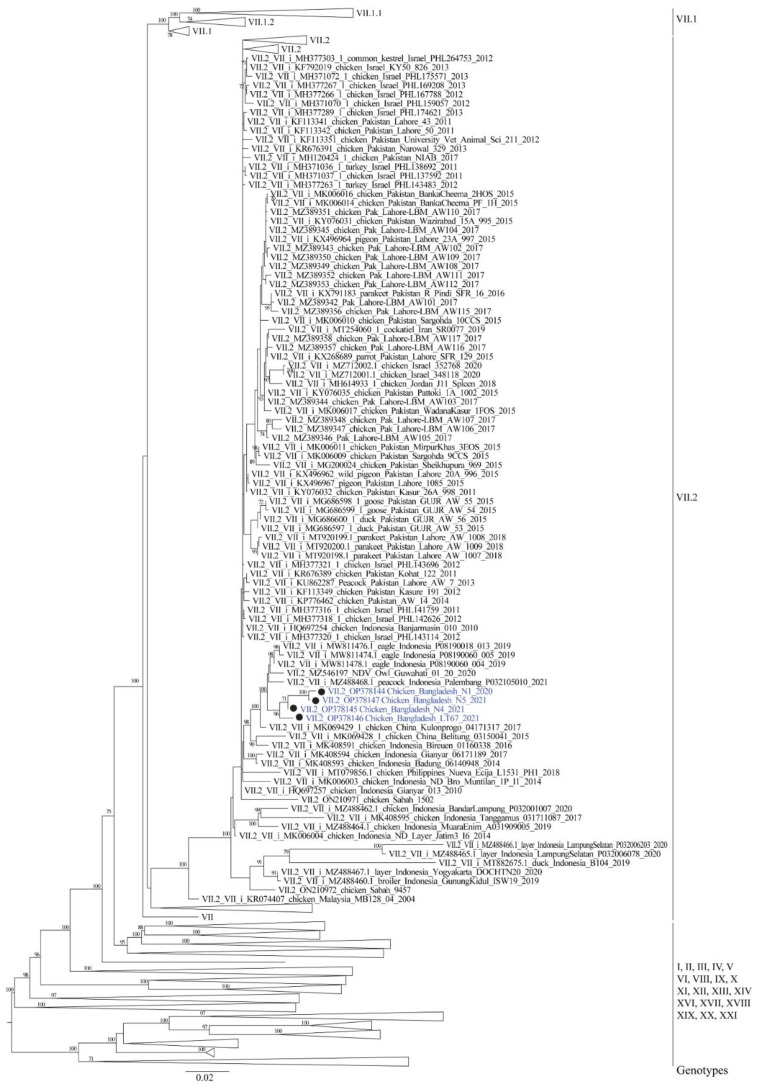
Phylogenetic analysis based on full-length nucleotide sequence of the fusion gene of Newcastle disease virus class II isolates (*n* = 2257). The evolutionary history was inferred by using RaxML [34] and utilizing the maximum likelihood method based on the general time-reversible model with 1000 bootstrap replicates. A discrete gamma distribution was used to model evolutionary rate differences among sites and the rate variation model allowed for some sites to be evolutionarily invariable. The tree is drawn to scale, with branch lengths measured in the number of substitutions per site. Bangladeshi isolates of present study are highlighted using filled circle (•). There were a total of 1653 positions in the final dataset. Only bootstrap values above 70 are shown.

The Basic Local Alignment Search Tool (BLAST) analysis of the Bangladeshi NDV isolates revealed 97.77% to 98.31% nucleotide identity (top five BLAST hits) to NDV isolates from India and Indonesia reported during the period of 2017–2021 (Owl/Guwahati/India/01/20, chicken/Kulonprogo/04171317/2017, Elang/Indonesia-Garut/P08190060-004/2019, Elang/Indonesia/Garut/P08190018-013/2019, and Peacock/Indonesia/Palembang/P032105010/2021). The phylogenetic analysis based on the full-length fusion protein gene sequences showed that the four studied NDV isolates group into genotype VII of class II NDV. The Bangladeshi isolates further clustered with sub-genotype VII.2 viruses (Figure 1 and Supplementary Figure S2) in the constructed full fusion gene class II phylogenetic tree and were designated as members of this NDV sub-genotype. Confirming the nucleotide distance analysis, the four Bangladeshi sub-genotype VII.2 isolates formed a monophyletic branch with the NDV isolates from India and Indonesia described above and several additional viruses from Indonesia, China, and the Philippines isolated between 2014 and 2021 (Figure 1 and Supplementary Figure S2).

To obtain a better insight into the genetic diversity of NDV circulating in Bangladesh, we further analyzed 45 fusion protein gene sequences of NDV from Bangladesh available in GenBank, including the four isolates studied here. Partial nucleotide sequences (203 nucleotides) were used in this analysis as full-length fusion gene sequences were available only for a few isolates. The analysis showed that three genotypes of NDV have been identified in Bangladesh between 2010 and 2021. While 29 NDV isolates from chickens and migratory birds belonged to genotype XIII, 12 isolates from pigeons clustered under the recently named genotype XXI.1.2. As described above, the four isolates investigated in the present study belonged to the genotype VII.2, which was never reported in Bangladesh heretofore (Supplementary Figure S3).

The four studied isolates had 97.7–99% nucleotide identity between them (Supplementary Table S1). Analysis of the intergroup nucleotide distances broadly followed the phylogenetic topology of the studied isolates. Estimates of evolutionary distance between different sub-genotypes of genotype VII NDV and the four Bangladeshi isolates is presented in Table 2. The three sub-genotypes of genotype VII viruses had 5.6–9.5% nucleotide distance between them. The four studied isolates from Bangladesh had an estimated 5.2% distance to sub-genotype VII.2 viruses, confirming their sub-genotype designation. Of note, these four isolates had a high nucleotide identity (98.3%, Supplementary Table S1) to a recent genotype VII.2 NDV isolate from a barn owl in India in 2020 (MZ546197/Owl/Guwahati/India/01/20). The viruses from Bangladesh and India were also closely related (98% to 99.2% nucleotide identity for most isolates, Supplementary Table S1) to NDV from Indonesia and China isolated between 2017 and 2021. In contrary, the newly studied Bangladeshi viruses were highly divergent (13.1% distant) from sub-genotype XIII.2 NDV previously reported to cause outbreaks in Bangladesh.

**Figure 2:**
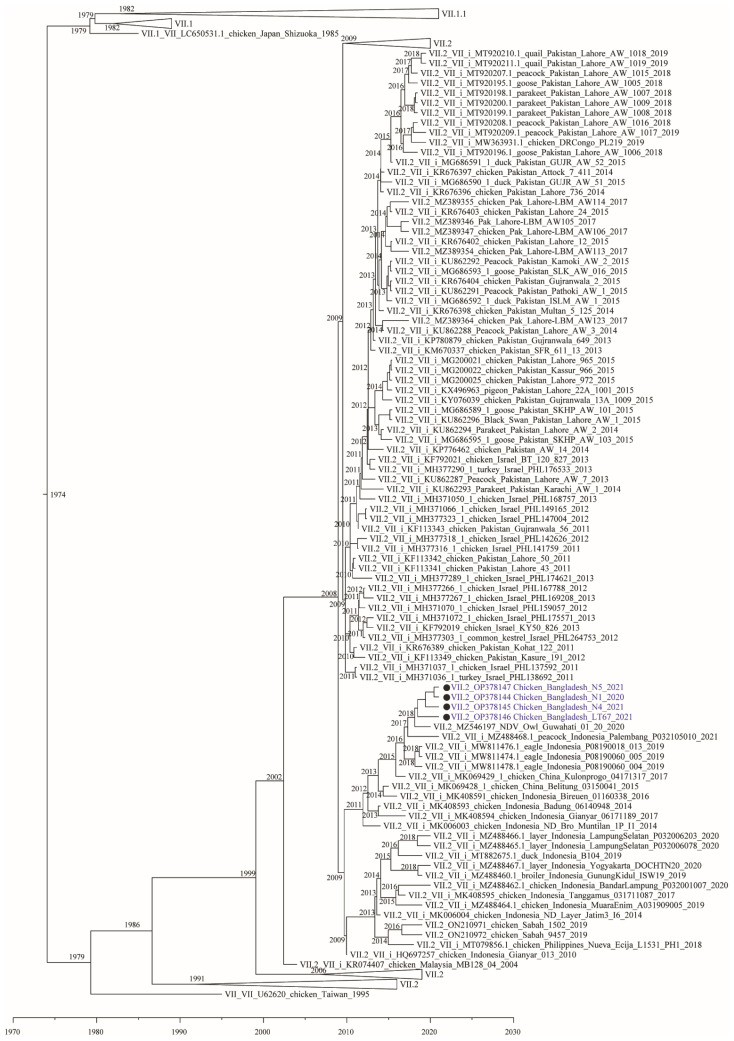
Maximum Clade Credibility (MCC) tree from Bayesian analysis utilizing a sub-set of all full fusion gene sequences (*n* = 1013) of genotypes VII. The analysis was conducted by the Bayesian Markov Chain Monte Carlo (BMCMC) method implemented in BEAST v1.10.4. The four Bangladeshi isolates are highlighted with filled circle (•).

To estimate the time to most recent common ancestor (tMRCA) between the studied isolates and related viruses, we used a Bayesian Markov Chain Monte Carlo approach. The Bayesian tree largely confirmed the topology of the Maximum Likelihood Analysis. The results provide evidence to suggest that the four Bangladeshi genotype VII.2 isolates together with the recent Indian isolate Owl/Guwahati/India/01/20 shared a common ancestor with isolates from Indonesia that existed around 2016–2017 (Figure 2).

### 3.2. Cleavage Site Analysis

The deduced amino acid sequences at the fusion protein cleavage site revealed presence of multiple basic amino acid residues (arginine/lysine) in all four studied NDV isolates with motif ^112^RRKKRF^117^. Such cleavage site is specific for virulent viruses based on the criteria utilized by the World Organisation for Animal Health to assess the virulence of NDV isolates [31].

### 3.3. Pathogenicity Testing

No co-infection with these influenza A virus, infectious bronchitis virus, and infectious laryngotracheitis virus was detected in any of the isolates. The N1, LT67 and N5 isolates showed MDT of 52.8 h, 36 h, and 52.8 h, respectively. Such MDT (<60) is typical for viruses that are velogenic for chickens [45]. Similarly, all three viruses had high ICPI values of 1.72, 1.67, and 1.61, respectively. Such high ICPI (>1.50) are typical for velogenic viruses [46]. The genetic and pathotype determinants/indices of the studied NDV isolates are shown in Table 3.

### 3.4. Experimental Infection in Chickens

**Figure 3:**
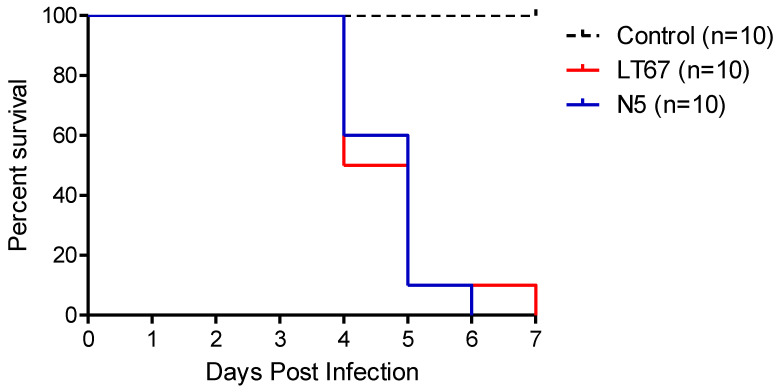
Survival curve in 35-days-old chickens experimentally inoculated with Newcastle disease virus isolates N5 and LT67.

All birds used in the experiments were found to be seronegative against NDV before the inoculation. The first clinical signs were observed at 3 DPI. Both the N5 and LT67 NDV isolates caused 100% morbidity and mortality in their respective groups. Signs of lethargy, respiratory distress, and diarrhea appeared at 3 DPI in both N5- and LT67-inoculated chickens. Mortality started at 4 DPI for both groups and all inoculated succumbed to infection by 6 DPI and 7 DPI, respectively (Figure 3, Supplementary Table S2). No clinical signs were observed in the sham-inoculated control group.

### 3.5. Gross and Microscopic Lesions

**Figure 4:**
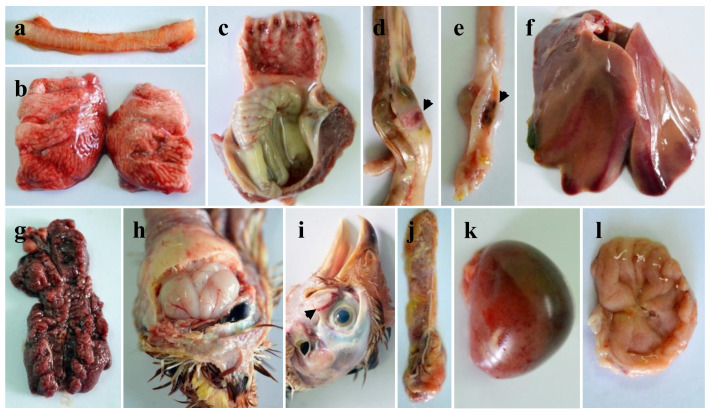
Gross pathological changes in chickens experimentally inoculated with LT67 isolates of NDV. (**a**) Hemorrhages in the trachea, (**b**) congestion in the lungs, (**c**) hemorrhages in the proventriculus, (**d**) hemorrhages in the intestines (button-like ulcers), (**e**) € hemorrhages in the cecal tonsils, (**f**) congestion in the liver, (**g**) severe congestion in the kidneys, (**h**) congestion in the brain, (**i**) hemorrhages in the Harderian glands, (**j**) hemorrhages and atrophy in the thymus, (**k**) congestion in the spleen, and (**l**) hemorrhages and slight atrophy in the bursa of Fabricius.

The gross and microscopic lesions in both N5- and LT67-inoculated chickens were broadly similar with slight variation in number of birds showing lesions (Figure 4a–l, Supplementary Table S3). Hemorrhages in the trachea and congestion of the lungs were observed in all inoculated chickens. Hemorrhages in the tip of the proventricular glands were found in seven and eight of the LT67- and N5-inoculated chickens, respectively. Button-like ulcers (three to six in number, eight birds) in the intestine were more frequently observed in the LT67-inoculated birds compared to the N5-inoculated birds (one to two in number, two birds). All birds from both inoculated groups showed hemorrhages in the cecal tonsils, congestion of the liver, severe congestion of the kidneys, and hemorrhages and slight atrophy of the bursa of Fabricius. Congestion of the brain was found in five and eight of the LT67 and N5-inoculated birds, respectively. Hemorrhages in the Harderian glands, hemorrhages and atrophy in the thymus and spleen were also found in most of the inoculated birds. Hemorrhages in the myocardium were present in two of the LT67-inoculated birds. No gross lesions were observed in any of the sham-inoculated birds of the control group.

**Figure 5:**
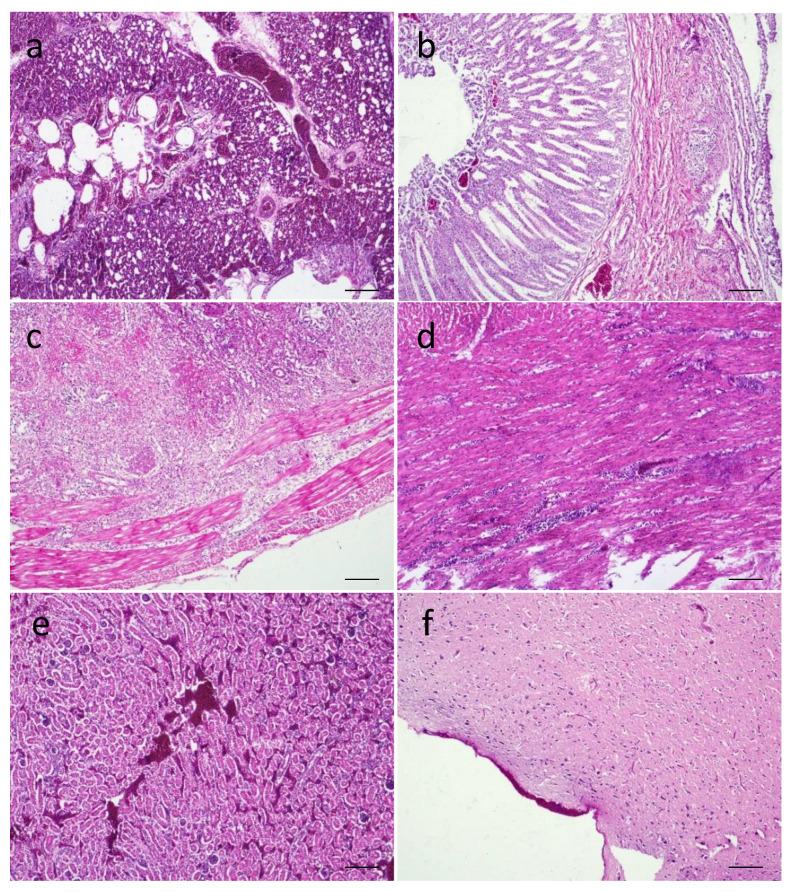
Microscopic changes in the tissues of LT67-inoculated chickens. (**a**) Section of lungs showing congestion and collapsed alveoli, (**b**) section of proventriculus showing focal proventriculitis with congestion and sloughing of epithelial cells, (**c**) section of intestine showing transmural enteritis, (**d**) section of heart showing focal myocarditis, € (**e**) section of kidney showing severe congestion and necrosis, and (**f**) section of brain showing congestion in the meninges. H&E stain. Bar (=50 μm) indicates magnification.

**Figure 6:**
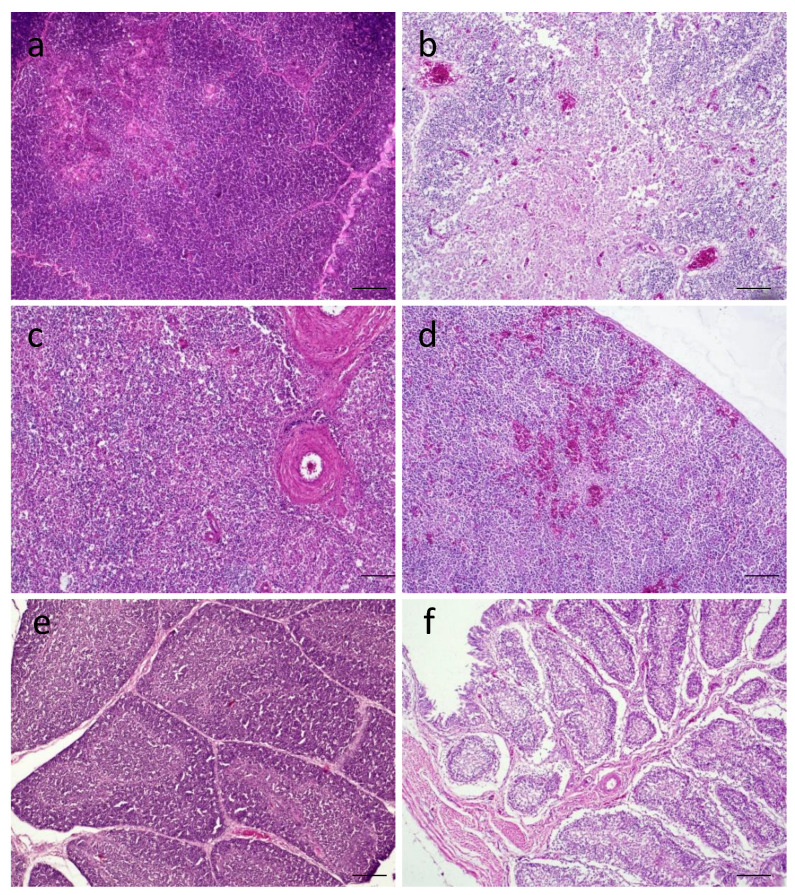
Histopathological changes in tissues of LT67-inoculated chickens. (**a**) Section of thymus of control chickens showing normal histology, (**b**) section of thymus of inoculated chickens showing congestion and multifocal lymphoid depletion, (**c**) section of spleen of control chickens showing normal histology, (**d**) section of spleen of inoculated chickens showing congestion in the parenchym€a, (**e**) section of bursa of Fabricius of control chickens showing some age-dependent depletion of lymphocytes in the bursal follicles, and (**f**) section of bursa of Fabricius of inoculated chickens showing severe lymphoid depletion leaving empty bursal follicles. H&E stain. Bar (=50 μm) indicates magnification.

The microscopic changes in the inoculated birds are presented in Figure 5 and Figure 6. Congestion and sloughing of epithelial layer in the trachea, severe congestion, hemorrhages and collapsed alveoli in the lungs were present (Figure 5a). Focal proventriculitis with congestion and sloughing of epithelial cells in the proventriculus (Figure 5b), necrotizing transmural enteritis with mononuclear infiltration (Figure 5c), loss of villi epithelium and accumulation in the lumen, blunting of villi and fusion of villi in the intestines were found. Focal myocarditis (Figure 5d) in the hearts of two birds was observed. In the kidneys, severe congestion and necrosis with slight infiltration (Figure 5e) and tubular nephritis were found. Severe congestion in the meninges was found in the brains (Figure 5f). Congestion, fatty changes, multifocal and portal hepatitis were observed in the livers.

Among various lymphoid tissues, there was congestion with multifocal depletion of lymphocytes in the thymus (Figure 6b), congestion in the spleen (Figure 6d), and severe lymphoid depletion in the bursa of Fabricius (Figure 6f). The control birds had normal histologic architecture in these lymphoid tissues (Figure 6a,c,e).

## 4. Discussion

Here, we report the full-length fusion gene coding sequences of four NDV isolated in Bangladesh from recent field outbreaks in 2020 and 2021. The isolates were identified as members of the sub-genotype VII.2 of class II NDV. This is the first report of viruses from this sub-genotype from Bangladesh. Pathogenicity testing of three of the isolates indicated high virulence. This was further confirmed by experimental infection of chickens. The observed clinical signs, and gross and microscopic lesions were consistent with velogenic nature of the viruses.

Here, we present the first complete fusion gene coding sequences of genotype VII from Bangladesh. Newcastle disease virus is endemic in poultry in Bangladesh. Outbreaks of the disease are associated with respiratory infections and high mortality in commercial flocks as well as backyard poultry in the country [23,25,47,48]. However, previous genotypic and pathotypic studies revealed the circulation of velogenic NDV belonging to genotype XIII.2 in chickens [27,28,29] and genotype XXI.1.2 in pigeons [30] in Bangladesh. Our recent study suggested the endemic circulation of sub-genotype XIII.2 in Southcentral Asia and further genetic diversification and establishing of these viruses in Bangladesh and neighboring India [27]. Heretofore, genotype VII NDV has not been reported in Bangladesh. A single NDV of genotype VII.2 was recently reported from a barn owl in Northeast India in 2020 [13]. Of note, the Bangladeshi genotype VII.2 NDV isolates had high nucleotide identity (98.2% to 98.8%) to the Indian genotype VII.2 isolate Owl/Guwahati/India/01/20. The molecular clock analysis estimated that the viruses evolved from a common ancestor that existed around 2016–2017. It is unclear if the reported genotype VII.2 NDV from Bangladesh and India evolved locally after initial introduction or evolved somewhere else and were separately introduced in the area. Obtaining additional full fusion gene sequences from historical isolates in the region will help to elucidate if other genotype VII viruses have been present before and around 2020.

The high genetic similarity (up to 99.2%) of the Bangladeshi and Indian genotype VII.2 NDV isolates with Indonesian and Chinese NDV suggests recent transboundary spillover from Southeast or East Asia to Central Asia. It is unlikely that the viruses from Bangladesh and India evolved from the sub-genotype VII.2 viruses that were previously introduced from Southeast Asia to Pakistan and the Middle East around 2011–2012 [21]. Those older viruses had less than 95% nucleotide identity to the newly isolates viruses from Bangladesh and India. Furthermore, it appears that two distinct lineages of sub-genotype VII.2 NDV are becoming established in Central Asia. The recent viruses from Bangladesh, Indonesia, India, and China form a monophyletic branch within sub-genotype VII.2 and are clearly separated from the viruses initially introduced to Pakistan and Israel more than a decade ago and further spread to several continents.

Newcastle disease viruses of genotype VII.2 are responsible for the fifth NDV panzootic [21] and continue to circulate and spread. Although originated in Malaysia and Indonesia, the genotype VII.2 NDV have been reported across continents, affecting bird populations across Asia, the Middle East, Europe, and Africa [10,13,14,15,16,17,18,19,20]. In Southeast Asia, the genotype VII.2 NDV were previously reported in Pakistan [19,21,49], where the viruses from this sub-genotype have become endemic. The long distance spread of NDV has been reported by the migratory birds [50] or through unintentional human interventions [51]. A study from Turkey reported circulation of the genotype VII.2 amongst wild birds and suggests the intercontinental spread of the virus through the migratory birds [20], which remains to be confirmed by further evidence. Locally in Bangladesh, many small and medium scale poultry farms are located close to households and have insufficient biosecurity practices. Traffic between backyard poultry and commercial flocks is frequent and backyard poultry often enter commercial farms grounds due to lack of structural barriers or fences. In addition, the long-distance movement of birds due to trade and the abundance of active live bird markets in the country likely contribute to virus transmission and further complicate the control of the disease.

The viruses from sub-genotype VII.2 continue to cause outbreaks in vaccinated chicken populations. It has been previously reported that these viruses cause outbreaks in vaccinated flocks with varying morbidity (up to 100%) and mortality (up to 50%) [52]. As ND vaccines do not provide sterilizing immunity, vaccinated birds get infected and shed challenge virus [53,54,55]. However, under experimental conditions, most vaccines provide protection from clinical signs and mortality, even after challenge with a high dose of velogenic NDV [56,57]. It is disputable if sub-genotype VII.2 viruses cause clinical signs and mortality in vaccinated flocks due to high genetic distance to conventionally utilized vaccines (commonly from genotypes I and II). This hypothesis needs to be supported by further evidence in addition to genetic analysis as experimental studies show that even though shedding is increased in birds infected with heterologous virus compared to those infected with homologous virus, birds are well protected in either scenario [53,54,55,56]. Other factors that can negatively impact vaccination and result in outbreaks in vaccinated flocks are co-infection with immunosuppressive organisms, presence of maternal antibodies which interferes with the development of active immunity, and sub-optimal vaccination practices, among others [57].

The standard pathogenicity testing revealed the velogenic nature of the genotype VII.2 NDV isolates from Bangladesh. To further study the pathogenic characteristics of these viruses, we inoculated 35-days-old seronegative chickens with two of the isolates (LT67 and N5) by using the natural routes of infection. Both viruses induced early clinical signs starting at 3 DPI and produced 100% morbidity and mortality in chickens, confirming the velogenic type of the viruses. The massive necro-hemorrhagic changes in different visceral organs of the inoculated birds determined the velogenic viscerotropic nature of the studied NDV [58,59,60]. At histopathology, massive necrotic changes were observed in different visceral organs of the inoculated birds. Besides, there was marked necrosis and depletion in different lymphoid organs of the inoculated birds. These changes are consistent with previously reported studies [58,61,62]. Of note, focal myocarditis with mononuclear infiltration was found in two of the genotype VII.2 NDV-inoculated birds in this study. A previous study with genotype VII.2 (former VIIi) also reported epicarditis and myocarditis marked by myocardial degeneration, and necrosis, edema, with mononuclear cell infiltration in the heart and the viral antigen was detected in the heart of the affected chickens [63].

## 5. Conclusions

We demonstrated for the first time the circulation of velogenic NDV belonging to the sub-genotype VII.2 in poultry in Bangladesh. The phylogenetic analyses suggest the establishment of two separate lineages of sub-genotype VII.2 NDV with a recent introduction of viruses in the region. This study confirms the capacity of sub-genotype VII.2 viruses to cause outbreaks in vaccinated birds and the mechanism for this remains to be clarified. The generated data provide comprehensive characterization and further knowledge on viruses circulating in Central Asia and will facilitate future studies of the genetic diversity of NDV.

## Figures and Tables

**Table 1:** Background information data of four Newcastle disease suspected outbreaks in Bangladesh between 2020 and 2021.

ID	Date	Farm Type	Location	Age	Flock Size	Morbidity (%)	Mortality (%)	Last Vaccination	Duration of Clinical Signs Prior to Diagnosis
N1	30 September 2020	Broiler Breeder	Mymensingh	8 weeks	9000	80	10	LaSota at 4 weeks of age	16 days
N4	4 April 2021	Broiler Breeder	Panchagarh	28 weeks	9000	50	2	Clone 30 at 22 weeks of age	10 days
LT67	16 February 2021	Broiler	Mymensingh	3 weeks	1000	65	20	Clone 30 at 4 days of age	7 days
N5	24 May 2021	Broiler	Gazipur	3 weeks	2000	70	26	Clone 30 at 4 days of age	3 days

**Table 2:** Estimates of evolutionary distance between different sub-genotypes of class II genotype VII Newcastle disease viruses and the Bangladeshi isolates studied here.

Sub-Genotypes	No. of Base Substitutions per Site *			
	VII.1.1	VII.1.2	VII.2	BD-VII.2
VII.1.1	-			
VII.1.2	0.056	-		
VII.2	0.095	0.079	-	
BD-VII.2	0.103	0.092	0.052	-
BD-XIII.2	0.132	0.119	0.128	0.131

* The number of base substitutions per site from averaging over all sequence pairs between groups are shown. Analyses were conducted using the Maximum Composite Likelihood model [38]. The rate variation among sites was modelled with a gamma distribution (shape parameter = 1). The analysis involved 1022 nucleotide sequences. Codon positions included were 1st + 2nd + 3rd +Noncoding. All positions containing gaps and missing data were eliminated. There were a total of 1653 positions in the final dataset. Evolutionary analyses were conducted in MEGA7 [37]. BD = Bangladesh.

**Table 3:** Genotypic and pathotypic indices of NDV isolates analyzed in this study.

Isolate	Accession No.	Genotype	MDT (h)	ICPI	Fusion Protein Cleavage Site	Pathotype
N1	OP378144	VII.2	52.8	1.72	^112^RRKKRF^117^	Velogenic
N4	OP378145	VII.2	N.D.	N.D.	^112^RRKKRF^117^	Virulent *
LT67	OP378146	VII.2	36	1.67	^112^RRKKRF^117^	Velogenic
N5	OP378147	VII.2	52.8	1.61	^112^RRKKRF^117^	Velogenic

Note: N.D.: Not done; ICPI: intracerebral pathogenicity index; MDT: Mean embryo death time; h: hours; * Only virulence but not pathogenicity can be inferred per fusion protein cleavage site.

## Data Availability

All authors agree that the data presented in this study are openly available through MDPI publisher platform or others without any restriction.

## Supplementary Materials

The following supporting information can be downloaded at: https://www.mdpi.com/article/10.3390/v14122627/s1, Figure S1: Map of Bangladesh showing the locations of the farms from which Newcastle disease viruses studied here were isolated. G = Gazipur, M = Mymensingh, and P = Panchagarh; Figure S2: Phylogenetic analysis based on full-length nucleotide sequence of the fusion gene of Newcastle disease virus class II isolates (*n* = 2257). The evolutionary history was inferred by using RaxML [34] and utilizing the maximum likelihood method based on the general time-reversible model with 1000 bootstrap replicates. A discrete gamma distribution was used to model evolutionary rate differences among sites and the rate variation model allowed for some sites to be evolutionarily invariable. The tree is drawn to scale, with branch lengths measured in the number of substitutions per site. Bangladeshi isolates of present study are highlighted using filled circle. There were a total of 1653 positions in the final dataset. Only bootstrap values above 60 are shown; Figure S3: Phylogenetic analysis based on partial nucleotide sequence of the fusion gene of Newcastle disease virus class II isolates (*n* = 165) from Bangladesh. The evolutionary history was inferred by using RaxML [34] and utilizing the maximum likelihood method based on the general time-reversible model with 1000 bootstrap replicates. A discrete gamma distribution was used to model evolutionary rate differences among sites and the rate variation model allowed for some sites to be evolutionarily invariable. The tree is drawn to scale, with branch lengths measured in the number of substitutions per site. Bangladeshi isolates of present study are highlighted using (filled circle). There was a total of 203 positions in the final dataset. Only bootstrap values above 60 are shown; Table S1: Estimates of evolutionary distance between Bangladeshi Newcastle disease isolates of class II sub-genotype VII.2 and closely related isolates from India, Indonesia, and China.; Table S2: Morbidity and mortality of chickens infected with NDV isolated N5 and LT67; Table S3: Gross pathological changes in chickens experimentally infected with NDV isolates N5 and LT67 (number of birds having the lesions shown in parenthesis).
