# Supplementary material for: The First Report of a Virulent Newcastle Disease Virus of Genotype VII.2 Causing Outbreaks in Chickens in Bangladesh
Source: Viruses. 2022 Nov 25;14(12):2627. doi: 10.3390/v14122627 (PMC9788440; doi:10.3390/v14122627)
Supplement: Supplementary file 1 [file viruses-14-02627-s001.zip › Suppl. Table S1.pdf]

Supplemental Table S1. Estimates of evolutionary distance between Bangladeshi Newcastle disease isolates of class II sub-genotype VII.2 and closely related isolates from India, Indonesia, and China.

Isolate	No. of base substitutions per site*								
	N1	N4	LT67	N5	MZ546197	MZ488648	MW811474	MW811476	MW811478
Chicken/Bangladesh/N1/2020									
Chicken/Bangladesh/N4/2021	0.012								
Chicken/Bangladesh/LT67/2021	0.023	0.010							
Chicken/Bangladesh/N5/2021	0.004	0.010	0.020						
MZ546197/owl/Guwahati/01/2020	0.018	0.012	0.017	0.017					
MZ488468/peacock/Indonesia/2021	0.025	0.017	0.019	0.022	0.012				
MW811474/eagle/Indonesia/005/2019	0.025	0.017	0.019	0.022	0.012	0.014			
MW811476/eagle/Indonesia/013/2019	0.023	0.015	0.017	0.020	0.010	0.012	0.002		
MW811478/eagle/Indonesia/004/2019	0.020	0.012	0.015	0.017	0.008	0.009	0.004	0.002	
MK069429/chicken/China/04171317/2017	0.023	0.015	0.017	0.020	0.013	0.013	0.013	0.011	0.009

*The number of base substitutions per site from averaging over all sequence pairs between groups are shown. Analyses were conducted using the Maximum Composite Likelihood model (Tamura et al., 2004). The rate variation among sites was modelled with a gamma distribution (shape parameter = 1). Codon positions included were 1st+2nd+3rd+Noncoding. All positions containing gaps and missing data were eliminated. There were a total of 1662 positions in the final dataset. Evolutionary analyses were conducted in MEGA7 (Kumar et al., 2016).
