# Supplementary material for: The First Report of a Virulent Newcastle Disease Virus of Genotype VII.2 Causing Outbreaks in Chickens in Bangladesh
Source: Viruses. 2022 Nov 25;14(12):2627. doi: 10.3390/v14122627 (PMC9788440; doi:10.3390/v14122627)
Supplement: Supplementary file 1 [file viruses-14-02627-s001.zip › Suppl. Table S2.pdf]

Supplementary Table S2. Morbidity and mortality of chickens infected with NDV isolated N5 and LT67.

dpi	1			2			3			4			5			6			7		
Status	N	S	D	N	S	D	N	S	D	N	S	D	N	S	D	N	S	D	N	S	D
Control	10	0	0	10	0	0	10	0	0	10	0	0	10	0	0	10	0	0	10	0	0
N5	10	0	0	10	0	0	0	10	0	0	6	4	0	1	5	0	0	1	0	0	0
LT67	10	0	0	10	0	0	0	10	0	0	5	5	0	1	4	0	1	0	0	0	1

Note: dpi: days post infection; N: Normal; S: Sick; D: Dead.
