# Supplementary material for: The First Report of a Virulent Newcastle Disease Virus of Genotype VII.2 Causing Outbreaks in Chickens in Bangladesh
Source: Viruses. 2022 Nov 25;14(12):2627. doi: 10.3390/v14122627 (PMC9788440; doi:10.3390/v14122627)
Supplement: Supplementary file 1 [file viruses-14-02627-s001.zip › Suppl. Table S3.pdf]

Supplementary Table S3. Gross pathological changes in chickens experimentally infected with NDV isolates N5 and LT67 (number of birds having the lesions shown in parenthesis).

Group	Trachea	Lungs	Proventriculus	Intestine	Caecal tonsils	Liver	Kidney	Brain	Heart	Harderian gland	Thymus	Spleen	Bursa of Fabricius
Control (n = 10)	Normal (10)	Normal (10)	Normal (10)	Normal (10)	Normal (10)	Normal (10)	Normal (10)	Normal (10)	Normal (10)	Normal (10)	Normal (10)	Normal (10)	Normal (10)
N5 (n = 10)	Haemorrhages (10)	Congestion (10)	Haemorrhages at the tip of the glands (8)	Button-like ulcers, 1-2 in number (2)	Haemorrhages (10)	Congestion (10)	Severe congestion (10)	Congestion (8)	Normal	Haemorrhages (8)	Haemorrhages and atrophy (4), haemorrhages (6)	Haemorrhages (2), Slight atrophy (8)	Haemorrhages and atrophy (10)
LT67 (n = 10)	Haemorrhages (10)	Congestion (10)	Haemorrhages at the tip of the glands (7)	Button-like ulcers, 3-6 in number (8)	Haemorrhages (10)	Congestion (10)	Severe congestion (10)	Congestion (5)	Haemorrhages (2)	Haemorrhages (8)	Haemorrhages (3), atrophy (5)	Congestion (3), mottling (2), Slight atrophy (1)	Haemorrhages, slight atrophy (10)
