# Supplementary figures and images for: The First Report of a Virulent Newcastle Disease Virus of Genotype VII.2 Causing Outbreaks in Chickens in Bangladesh
Source: Viruses. 2022 Nov 25;14(12):2627. doi: 10.3390/v14122627 (PMC9788440; doi:10.3390/v14122627)

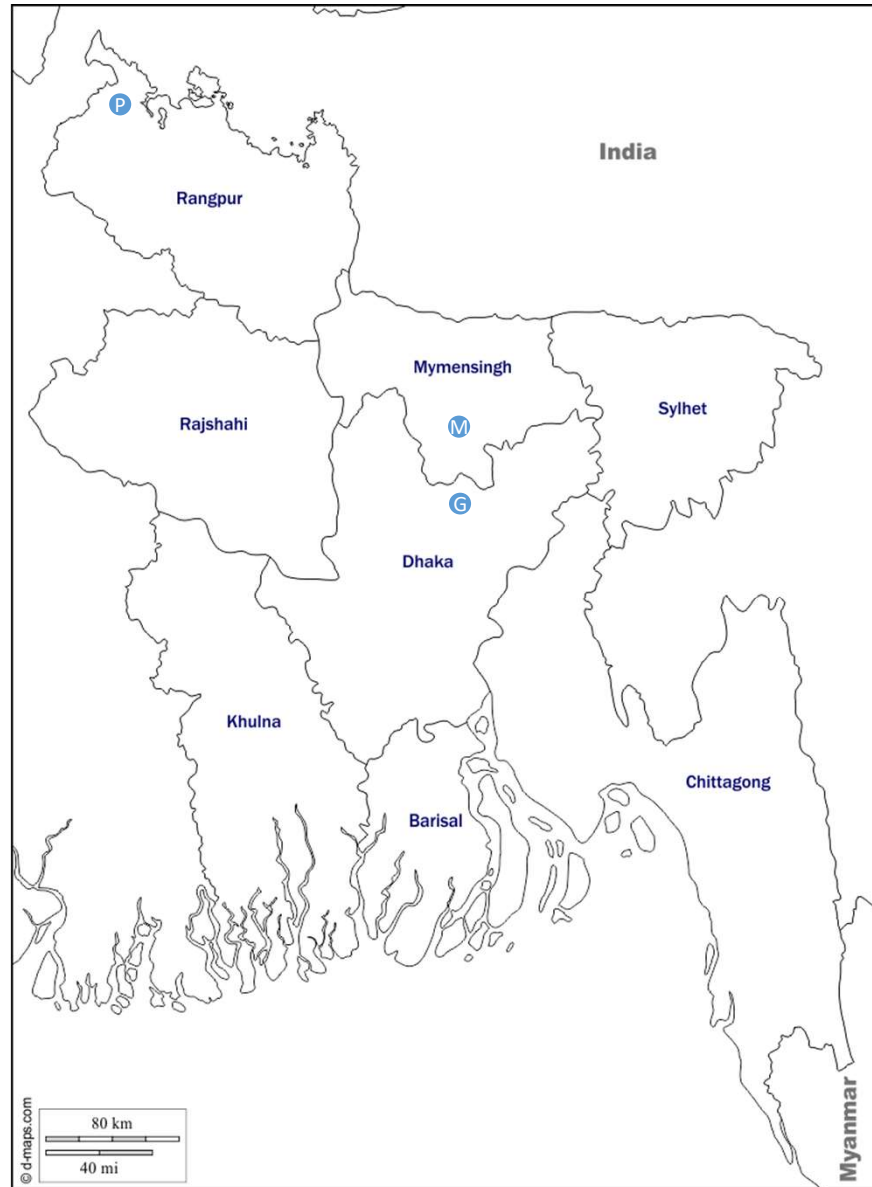

Supplement: Supplementary file 1 [file viruses-14-02627-s001.zip › Suppl. Fig. S1.pdf]

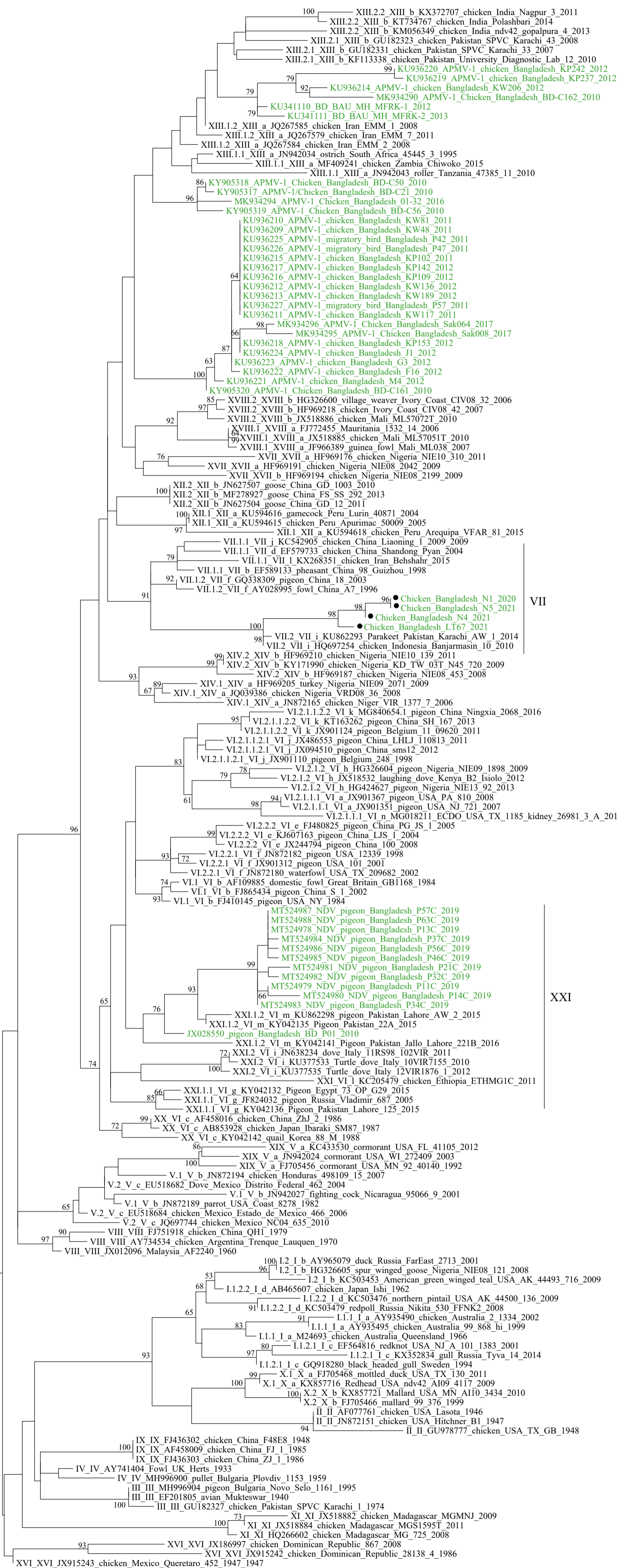

XIII

VII

XXI

0.04

Supplement: Supplementary file 1 [file viruses-14-02627-s001.zip › Suppl. Fig. S3.pdf]
