# Supplementary material for: The First Report of a Virulent Newcastle Disease Virus of Genotype VII.2 Causing Outbreaks in Chickens in Bangladesh
Source: Viruses. 2022 Nov 25;14(12):2627. doi: 10.3390/v14122627 (PMC9788440; doi:10.3390/v14122627)

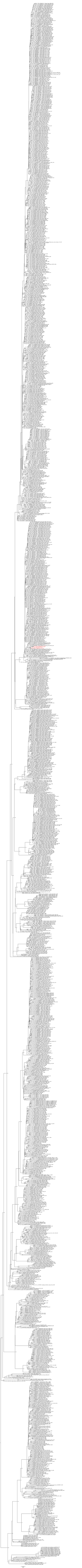

Supplement: Supplementary file 1 [file Suppl. Fig. S2.pdf › Suppl. Fig. S2.pdf]
